# A novel geometrical planning method to restore knee joint obliquity in double-level osteotomies

**DOI:** 10.1007/s00402-023-04997-6

**Published:** 2023-07-28

**Authors:** Marcello Capella, Luigi Sabatini, Francesco Bosco, Luca Barberis, Fortunato Giustra, Salvatore Risitano, Daniele Camazzola, Alessandro Massè

**Affiliations:** Department of Orthopaedics and Traumatology, University of Turin, CTO, Via Zuretti 29, 10126 Turin, Italy

**Keywords:** Double-level osteotomy, Joint line obliquity, Knee osteotomy, Limb deformity, Coronal deformity, Digital planning, Software, Method

## Abstract

**Purpose:**

Precise preoperative planning is mandatory when a double-level osteotomy (DLO) is required to correct a severe knee deformity. Literature does not report a validated planning method regarding DLO that could be performed directly on digital radiographs using simple measurement tools. This study aims to validate a novel DLO planning method called New Mikulicz-Joint Line (NM-JL) based on essential measurement tools, in which the correction angles are induced by the predicted post-operative joint line obliquity (JLO).

**Methods:**

Twenty-three patients who satisfied the inclusion criteria were enrolled. NM-JL planning method was performed using basic measurement tools to detect corrective angles and gaps. The correction was then simulated using a Virtual Segmentation Software method to obtain the osteotomy fragments. Both planning procedures were performed independently and later repeated by two orthopaedic surgeons to assess the inter and intra-observer reliability.

**Results:**

The intraclass correlation coefficient (ICC) regarding corrective angles and gaps showed a significant positive correlation between the values determined using the two procedures by both raters (*p* < 0.05). Pearson’s correlation analysis revealed a significant correlation between the measured results of the two planning methods. (*p* < 0.05). Finally, the Bland–Altman analysis showed an excellent agreement (*p* < 0.05) for all measurements performed.

**Conclusions:**

The NM-JL method showed high values of intra and inter-rater reliability. The procedure is built up starting from the predicted value of post-operative joint line obliquity, allowing to maintain this parameter fixed. Other advantages include the quickness, adaptability, and possibility to be performed on any Digital Imaging and Communication in Medicine (DICOM) viewer.

**Level of evidence:**

Level IV.

**Supplementary Information:**

The online version contains supplementary material available at 10.1007/s00402-023-04997-6.

## Introduction

Osteotomies around the knee have been performed to redistribute the load on the intact compartment to reduce pain symptoms and delay or avoid the need for knee arthroplasty [1]. Historically, a varus knee was primarily related to a tibial deformity and exclusively corrected through a high tibial osteotomy (HTO) [2–5]. Similarly, a valgus malalignment was treated through a distal femoral osteotomy (DFO), ascribing the deformity at the distal femur [6, 7]. However, recent studies have shown that varus malalignment may result from tibial, femoral, or combined deformity [8–11]. Furthermore, in some patients, the coronal malalignment may partially or entirely belong to intra-articular wear or ligamentous loosening instead of pure bone deformity [5, 9]. Therefore, as described by Paley et al. [12], corrective knee osteotomies should be performed at the site of the original deformity. The surgical restoration of a proper coronal axis may result in a new bony deformity that leads to excessive joint line obliquity (JLO) [13] and increased shear forces on the articular surface [14] that may jeopardise the results. Therefore, in some patients, a double-level osteotomy (DLO), which is a combination of an HTO and a DFO, may be necessary to correct the deformity and realign the knee joint obliquity [13, 15–18]. A meticulous preoperative planning is mandatory; otherwise, it may adversely affect the osteotomy procedure’s functional outcome and survival rates, increasing the risk of later total knee arthroplasty [15, 17].

Regarding HTO and DFO, different planning methods are validated on standing long-leg radiographic views, such as the Dugdale and Miniaci methods [19, 20]. On the other hand, DLO planning is currently performed exclusively with advanced software using virtual segmentation [15, 16, 21]. At the same time, no validated methods allow accurate planning with simple tools of any Digital Imaging and Communication in Medicine (DICOM) viewer software that could be considered reliable and reproducible.

This study aims to validate the New Mikulicz-Joint Line (NM-JL) method as a DLO planning method. This procedure detects corrective angles and gaps on the femoral and tibial sides, distributing the correction on each level and preserving the physiologic JLO. It could be performed directly on full-length standing AP view X-ray using simple measurement tools available on any DICOM viewer, such as angles, lines, and ruler. The hypothesis is that the proposed NM-JL method is reproducible, reliable, and comparable to a software method based on virtual segmentation.

## Materials and methods

Between January 2020 and June 2022, a total of 23 patients were recruited in our department who satisfied all the radiological inclusion criteria for a DLO for a varus knee: a preoperative distal lateral femoral angle (LDFA) > 90° combined with a proximal medial tibial angle (MPTA) < 85°; an MPTA > 94° obtained from the planning of a single HTO; the need of a significant correction with > 10 degrees or > 15 mm gaps [15, 16, 21, 22]. In addition, exclusion criteria were advanced knee osteoarthritis KL grade 4, significant articular deformity with joint line convergence angle (JLCA) > 6°.

Radiological measurements and analysis were performed on a full-length AP digital radiograph. The digital radiographs were imported into TraumaCad^®^ TM 2.4 (Brainlab, Voyant Health Inc.), a digital planning software designed for medical imaging. The software can be used as a simple DICOM viewer with essential measurement tools such as angles, lines, and ruler, or it can provide digital planning, with the possibility to perform a virtual segmentation of osteotomy fragments simulating the correction. The digital radiograph can be modified in both cases, generating correction angles, and opening or closing gaps to provide the following DLO procedure.

Following a reported method described by Jacquet et al. [23], a mechanical and anatomical angle analysis, according to Paley, was performed [12, 24]. The HKA angle, MPTA, LDFA, and JLCA were measured for each knee, obtaining reproducible and consistent values for each patient and an associated marginal error of fewer than 2 mm (mm) and less than 1 degree. The reference values based on studies in the literature were: LDFA 85.8 ± 2.0, MPTA 85.6 ± 2.4, HKA 179.4 ± 2.6, JLCA 1.09 ± 0.9 [12, 25].

### NM-JL method

**Fig. 1:**
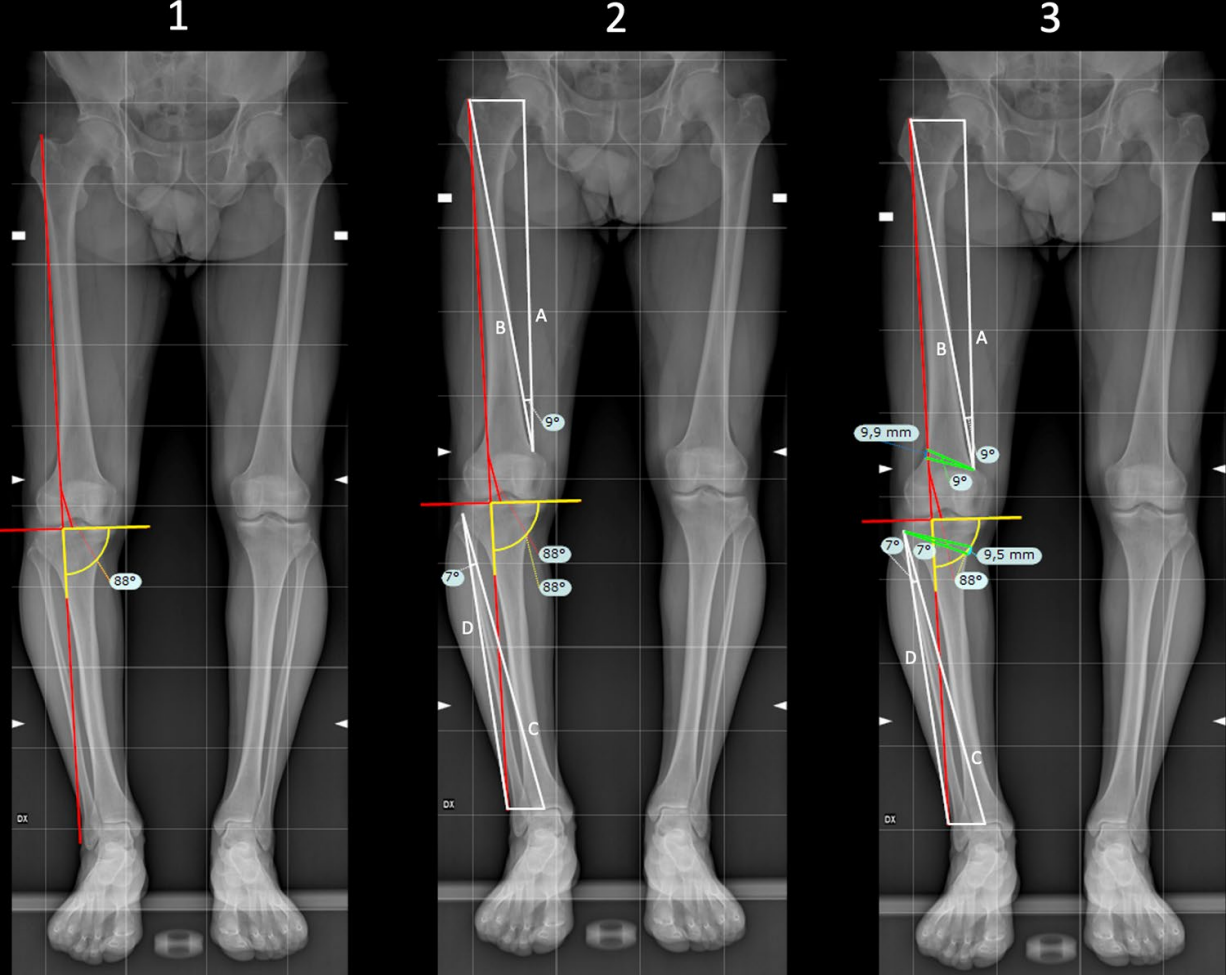
Bilateral weight-bearing full-length AP digital radiograph for DLO planning according to the authors’ proposed method. **1.1** A 88 degrees of NM-JL value is established. Determine a correction point at the knee joint line at 62.5% of the tibial plateau width measured from the medial tibial cortex. Draw the new loading axis passing through the correction point defined. **1.2** The hinge point for the osteotomies was determined. At the femoral level, an A-line connects the femoral hinge point and the hip centre. At the same time, a B-line connects the femoral hinge point and the intersection point of the new loading axis at the femoral level. A C-line connects the tibial hinge point and the centre of the ankle joint at the tibial level. Finally, a D-line connects the tibial hinge point and the intersection point of the new loading axis at the tibial level. **1.3** The angle between these lines represents the femoral correction angle (the green angle at femoral level) and the tibial correction angle (the green angle at tibia level.) The osteotomies’ millimetre open and closure gaps were measured (blue lines at femoral and tibial levels)

**Fig. 2:**
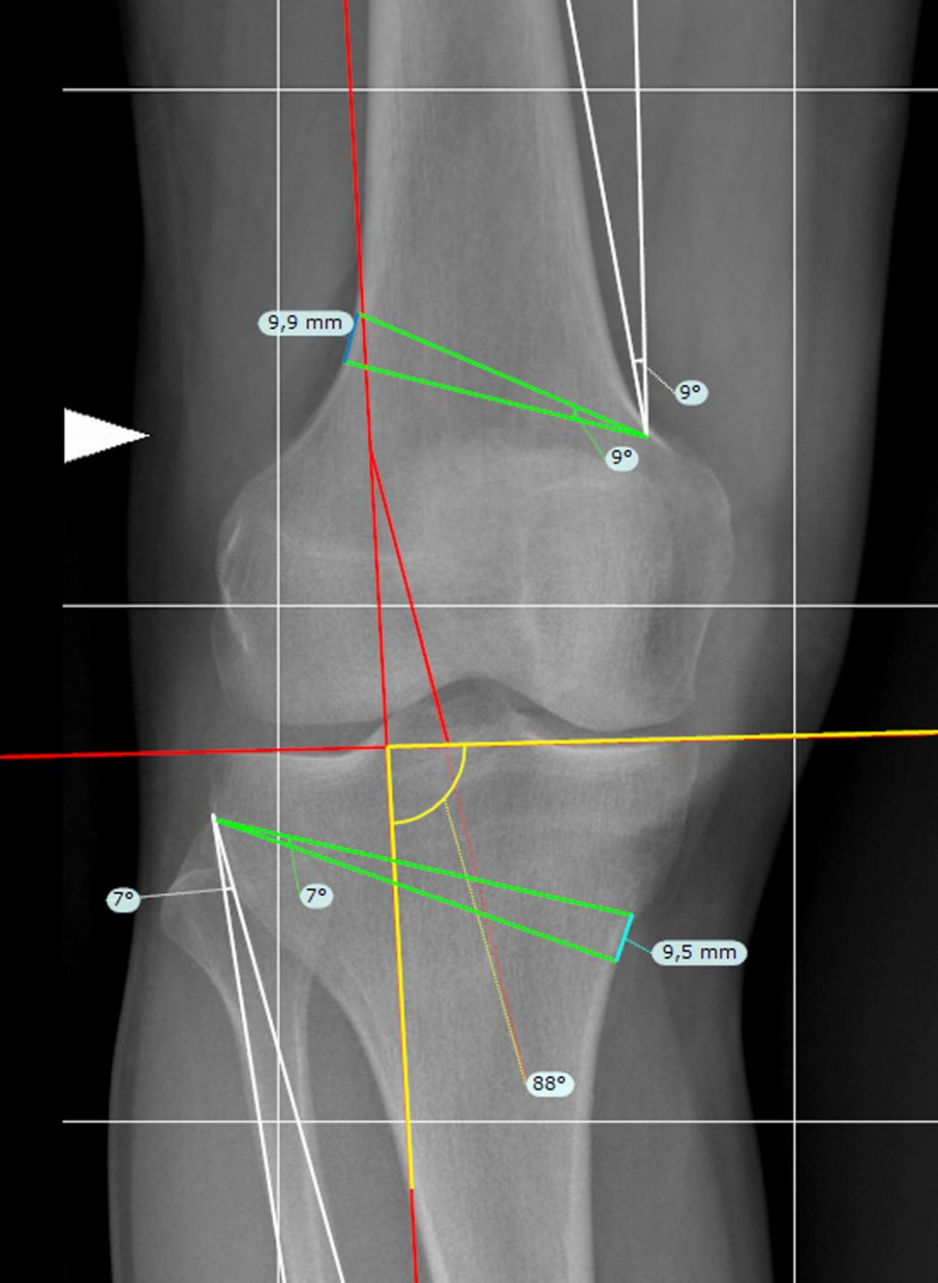
Magnificated images of the proposed planning by the authors to better illustrate angles and correction gaps of the osteotomies

The NM-JL geometrical planning method (Figs. 1 and 2) was performed in the following manner.

- The recorded measurements were calibrated using TraumaCad^®^ software with the previously defined 100 × 100 mm reference square. The width of the tibial plateau was measured by drawing an unbroken horizontal line directed towards the medial and lateral margins, excluding osteophytes findings.
- The correction point at the knee joint line has to be set depending on the clinical case. In our study, for demonstrative purposes, the correction point was set at 62.5% of the tibial plateau width measured from the medial tibial cortex.
- A vertical line, representing the new Mikulicz line, intersecting the tangent of the tibial plateau through the chosen correction point and subtending a medial lower angle of a determined value was drawn (Fig. 1, 1.1). This angle, called NM-JL, depicted the post-operative JLO of the realigned knee and was set at 88 degrees in our study.
- The hinge point for the osteotomies was determined [26]. The tibial hinge point was positioned approximately 1.5 cm below the lateral joint line near the head of the fibula, while the femoral hinge point was positioned terminating near the adductor tubercle as described by other authors [27, 28] (Fig. 1, 1.2).
- An A-line was drawn from the femoral hinge point towards the centre of the hip. A B-line was drawn from the femoral hinge point towards the new femoral load axis point of intersection with a line parallel to the ground starting from the centre of the femoral head. The angle between these two lines represents the femoral correction angle (Fig. 1, 1.2).
- A C-line was drawn from the tibial hinge point towards the centre of the ankle joint. A D-line was drawn from the tibial hinge point towards the intersection of the new tibial loading axis with a line parallel to the ground from the centre of the ankle joint. The angle between these two lines represents the tibial correction angle (Fig. 1, 1.2).
- The osteotomy lines were then drawn. Specifically, the femoral osteotomy line (Fig. 1, 1.3), green line on the femur, was oriented from the lateral cortex to the medial terminating near the adductor tubercle and from proximal to distal forming an angle of approximately 20 degrees with the femoral perpendicular axis. The tibial osteotomy line, green line on the tibia, was defined by drawing a straight line from the medial cortex, approximately 4 cm below the knee joint line above the pes anserinus, to the previously described lateral hinge point at forming an angle of roughly 20 degrees with the tibial perpendicular axis [27, 28] (Fig. 1, 1.3).
- At the femoral level, the distal arm of the angular instrument centred on the femoral hinge point was then swung proximally for an angle equal to the previously calculated correction value. Both lines thus obtained (Fig. 2), green lines, were extended until they intersected the lateral femoral cortex. The distance between the two lines at the intersection points with the lateral cortical was recorded as the millimetric closure gap at the femoral level. On the other hand, at the tibial level, the angular instrument’s distal arm centred on the tibial hinge point was swung proximally for an angle equal to the correction value calculated previously. Thus, both obtained, green lines, were prolonged until they intersected the medial tibial cortex. The distance between the two lines at the intersection points with the medial tibial cortex was recorded as the millimetric open gap at the tibial level, blue lines at femoral and tibial levels (Fig. 2).

### Virtual segmentation software (VSS) method

**Fig. 3:**
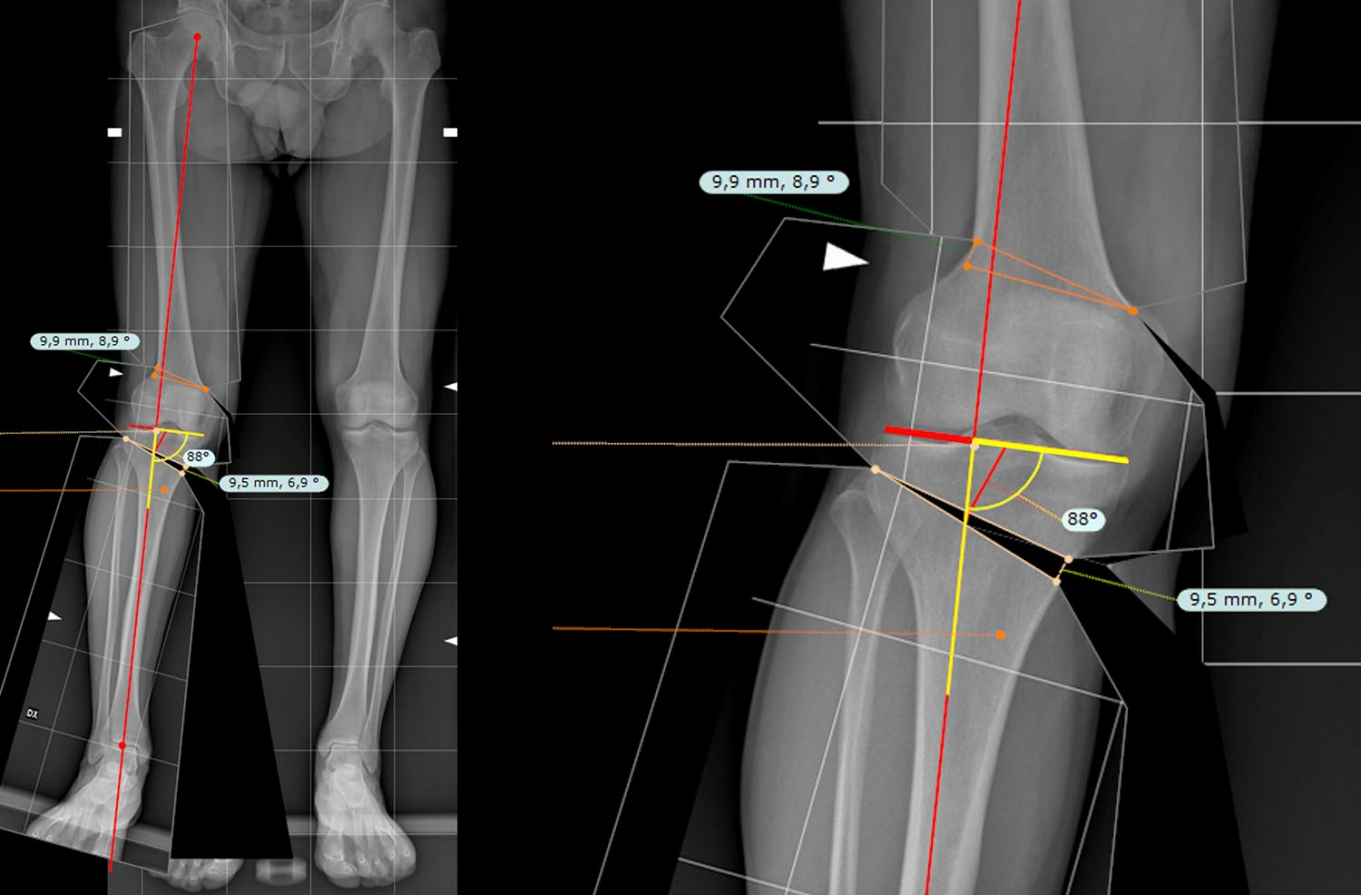
Using the proposed method by the authors, the NM-JL angle of 88° was simulated on the TraumaCad^®^ using the Virtual Segmentation Software (VSS) method to check whether the same gap values and femoral and tibial angles were obtained

- The full-length standing AP view radiograph was calibrated on TraumaCad^®^ using the 100 × 100 mm reference square.
- Afterwards, using the instrument “Limb Alignment Analysis”, the anatomical landmarks were selected to obtain the mechanical axis and angles of the lower limb, including LDFA, MPTA, JLCA and the weight-bearing line.
- An unbroken line was drawn tangent to the tibial plateau, and the proximal tibia width was then measured, excluding the osteophytes.
- The new load-bearing point was established at 62.5% of the tibial width measured from the medial tibial cortex.
- The hinge points were then determined in the same manner described above.
- The virtual segments were manually rotated, creating a medial open wedge for the tibia and a lateral closing wedge for the femur. Segments were progressively rotated until the weight-bearing axis intersected the tibial plateau at 62.5% and simultaneously formed a medial angle of 88 degrees with its tangent (Fig. 3).
- The obtained correction angles and the open/close gaps on the tibia and femur were recorded and collected.

### Data collection

Data for the intra- and inter-rater reliability studies were collected from 23 patients. On preoperative radiographs, two authors (LS and MC) independently determined the correction angles and millimetric gaps for femoral and tibial osteotomy. These values were derived from both the NM-JL and the VSS method.

For all 23 cases, both trained raters performed the planning with the NM-JL method at time zero and after at least 30 days. Similarly, the VSS method was executed 60 days from the time zero and then repeated at 90 days. The correction angles values and millimetric gaps for femoral and tibial osteotomy, derived from both planning, were recorded and collected. The patients’ data were blinded, and the sequence of recordings was randomised for both planning methods.

### Statistical methodology

Data from the NM-JL method (at time zero and after 30 days) and the VSS method (after 60 days and after 90 days) were compared for each rater to assess intra-observer variability. In addition, measurements from the two planning methods were compared between the two raters for each series of planning (NM-JL at time zero and VSS after 60 days; NM-JL after 30 days and VSS after 90 days) to evaluate inter-observer variability. Intra- and inter-observer variabilities were calculated with intraclass-correlation-coefficient (ICC) using a two-way random model for continuous variables with absolute agreement. The ICC values ranged from 0.00 to 1.00, with solid reliability reported for values close to 1.00. Values below 0.5, between 0.5 and 0.75, 0.75 and 0.9, and above 0.90 indicated poor, moderate, reasonable, and excellent reliability, respectively. A 95% confidence interval (CI) of the estimated ICC was used.

Moreover, the Pearson's correlation coefficient and Bland–Altman diagrams compared the two planning methods. The first one was used for correlation analysis and thus to determine whether the values of the two analysed variables are associated. The correlation coefficient (*r*) to resolve this association was used. It is a number between − 1 and 1. Values close to 1 describe the relationship between the two variables perfectly. The Bland–Altman method was used to plot the difference between the correction angles and the millimetric gaps for both femoral and tibial osteotomy determined with the two methods. The limits of agreement (LoA) are defined as the mean difference ± 1.96 standard deviations (SD). If these limits do not exceed the maximum allowed difference between methods, the two methods agree and may be used interchangeably. Also, for the Pearson correlation coefficient and the Bland–Altman method, a 95% CI was considered to specify that the two methods do not disagree. All statistical analyses were performed using MedCalc software version 13, 2014 (MedCalc Software, Ostend, Belgium).

## Results

A total of 23 patients were enrolled in our study. The average age of included patients was 52.87 ± 5.41 years (range 41 to 62 years). There was a male predominance (*n* = 17, 73.9%). Regarding the main parameters of the radiographic deformity: the average HKA was 11.43 ± 2.33; the average MPTA was 81.52 ± 1.86; the average LDFA was 93.13 ± 1.49; the average JLCA was 4.05 ± 1.79. Demographic data and deformity analysis of patients enrolled in this study are reported in Table 1. The ICC regarding preoperative radiographs showed high agreement for all measurements in both software and raters. Intra-rater and inter-rater reliability analyses are described in Additional File 1.

**Table 1:** Main demographic characteristic and deformity analysis of patients enrolled in this study

Sample size sex		Sample size age, y.o.		Deformity analysis							
				HKA		MPTA		LDFA		JLCA	
M, *n*	F, *n*	Mean	SD	Mean	SD	Mean	SD	Mean	SD	Mean	SD
17	6	52.87	5.41	11.43	2.33	81.52	1.86	93.13	1.49	4.05	1.79

*y.o.* years old, *HKA* hip–knee–ankle angle, *MPTA* proximal medial tibial angle, *LDFA* distal lateral femoral angle, *JLCA* joint line convergence angle, *M* male, *n* number (of patients), *F* female, *SD* standard deviation

The intra-rater reliability analysis showed high agreement between the different measurements performed by the same rater, with both planning methods ranging from minimum ICC values of 0.977 (95% CI 0.9481–0.9905) to maximum ICC values of 0.9993 (95% CI 0.9982–0.9997).

The inter-rater reliability analysis showed high agreement between the two raters in all radiographic assessments compared with both planning methods, ranging from minimum ICC values of 0.9831 (95% CI 0.9604–0.9928) to maximum ICC values of 0.9995 (95% CI 0.9988–0.9998). In addition, Pearson’s correlation was used to compare the values obtained from the geometrical NM-JL and VSS methods. There was a significant positive correlation between the values determined using the two procedures by both raters (*p* < 0.05). Furthermore, the Pearson’s correlation analysis performed by measuring the same parameters at 30 days for the NM-JL method and 90 days for the VSS method by both raters revealed a significant correlation between the measured results of the two planning methods. (*p* < 0.05). Furthermore, the Bland–Altman analysis showed that the bias, in terms of percentage difference, was independent of the size of the correction. The Cis’ limits were represented for our data set by comparing the different evaluations. All measurements performed could be evaluated as excellent agreement (*p* < 0.05). The data analysed with the Bland–Altman analysis were reported in Additional File 2.

## Discussion

This study proposes and validates a new planning method for DLO based on simple geometrical measurements on a full-length standing AP radiograph. Method reproducibility, assessed with intra- and inter-rater reliability analysis, showed high agreement between observers regarding correction angles and millimetric opening/closing gaps. Furthermore, a remarkable agreement was obtained on the Bland–Altman analysis comparing our geometrical and virtual segmentation software-based methods.

Thorough deformity analysis and accurate preoperative planning are crucial for successful DLO surgeries, similar to HTO and DFO [27–29]. Previous studies on DLOs have used different software tools for planning realignment procedures [30–33]. However, no validated DLO planning method reported in the literature can be directly performed on full-length standing AP X-rays using simple measurement tools. The proposed planning method is not entirely novel but refers to the previously theoretical principles described by Miniaci and Jakob and the different planning methods and principles defined by Paley [12, 20, 24, 34]. The main advantage is that the proposed planning method is based on the predicted post-operative JLO, and the correction angles at the two sides are derived from this parameter and not the reverse. This goal is reached by introducing a new index of the post-operative JLO, the NM-JL angle. Furthermore, the proposed method could be applied to a basic DICOM viewer that should always be available even at the first clinical examination of the patient. Since the NM-JL planning method is based on simple measurement tools such as open or Cobb angle, line, and ruler available in any DICOM viewer, it can be employed even if a specific medical planning software is not accessible. Moreover, this new geometrical method provides correction angles and millimetric gaps without acquiring specific anatomical landmarks as the landmark-based software, saving time during the planning phase. To determine the final gaps, if a calibration tool is not included in the basic software, a 3% increment is usually suggested. A further feature of the NM-JL method, which was described in this paper with strict parameters and magnitudes to obtain a reproducible protocol for its validation, allows a wide range of variability: the proposed method is easily adaptable in terms of the amount of correction, because the load point on the tibial plateau can be chosen depending on the specific clinical case. In this study, according to Fujisawa et al. [35] and applying the osteotomy rules defined by Paley, the new load axis was set at 62.5% of the tibial plateau width, determining a slight overcorrection. Regardless, this value can be adapted starting from 50% of the tibial plateau width proposed by other authors, considering other issues such as the medial compartment cartilage thickness [36–38]. In clinical cases requiring higher amounts of unloading, this method allows equally allocating the overcorrection at the two levels for the abovementioned reasons. Also, the NM-JL angle could be freely suited according to the specific case. In our series, an 88-degree angle was set to maintain a slightly varus inclination of the joint line, but this value could be increased or decreased within narrow limits.

An increase in NM-JL angle corresponds to a higher tibial correction at the expense of the femoral one; in contrast, its decrease corresponds to a lower tibial correction favouring a higher femoral closure. However, the correction allocation within the two levels entails a precise effect on the JLO, which is visible and immediately valuable in acceptability. Some previous studies have already introduced the Mikulicz Joint Line angle (M-JL) as an index for JLO [22, 26, 39]. M-JL is currently described as the angle between the Mikulicz line and the bisector of JLCA [22, 26]. Compared to the angle between the joint line and the ground, it has the consistent advantage of being independent of the position of the leg in the coronal plane; M-JL is also more accurate than MPTA since the latter has a link with the JLO that HKA strongly influences.

The proposed method is based on anatomic landmarks in the preoperative X-rays and maintains a definite match to the post-operative scenario. We decided to use the tangent of the tibial plateau as the joint line representative: this is a constant landmark before and after the correction, while a high JLCA is intended to strongly decrease because of the leg realignment, resulting in the lack of a reasonable, predictable match between the bisector of the preoperative JLCA and the corrected knee. The value of the NM-JL angle can be modified during the planning based on the desired JLO and the corrections between the two levels. In this study, 88 degrees were always set as an NM-JL measure for demonstrative purposes compared to the VSS method. This value is considered the first choice in our real-life planning. There are some considerations to debate considering 87 degrees as the physiologic JLO standard (measured as an M-JL angle). The first issue is that higher values of post-operative M-JL angle are common and usually well-tolerated, while the literature is poor regarding lower values. Secondly, considering the variable amount of cartilage wear in the medial compartment, which contributes to the persistence of a post-operative JLCA higher than 2 degrees, the bony anatomy, usually considered a radiographic landmark, provides an overestimation of the valgus of the concrete weight-bearing tibial baseplate. Based on corrective angles and gaps allocation, the patient's characteristics, including physiologic issues such as age, body mass index (BMI) and comorbidity, as long as anatomical ones like patella height and limb length discrepancy, the NM-JL value is currently modulated within the range reported by the literature, preferably among 87 and 91 degrees [22, 26, 39].

In this series, we have not considered a correction for intra-articular deformity cause; being independent that is applied to the angles and gaps given by any chosen planning method, it was not strictly related to the aim of the study. Similar to the Dugdale and Miniaci methods for HTO [19, 20], also in our planning procedure, the soft tissue tension and the intra-articular deformity, depicted by a pathological JLCA angle [40], are intrinsically incorporated into the bony correction. In the same way, using the VSS method, the articular deformity is not considered, and to mitigate its detrimental effects, a specific centre of rotation of angulation (CORA) must be added at the joint line level before the automatic or manual rotation of the femoral and tibial segments. If the articular and soft tissue contribution to the overall deformity is not separately processed, a significant risk of overcorrection is present, which is of particular interest in the case of a more significant JLCA value [40].

For HTO, several authors have proposed mathematical models to predict and correct the JLCA to the tibial correction [40–43]. As with single-level osteotomies, for DLOs, in patients with high JLCA, it still remains debated where the post-operative correction axis should be set. Therefore, the JLCA influence should be considered when performing a DLO, but how to distribute the correction remains debated. Similar to Micicoi et al. for HTO [42], we routinely evaluate a soft tissue correction when JLCA > 2°. Usually, the global amount of the bony correction decreases after the articular deformity is deducted using the formula (JLCA-2)/2 [8]. Due to the nature itself of the planning, considering the tangent to the tibial plateau as a starting point and a reference of the joint line, the NM-JL method tends to attribute the intra-articular deformity to the femur, and so this quote should be entirely subtracted from the femoral correction angle.

This study has several limitations. The study was conducted with a restricted number of cases, which could be explained considering that the interest in DLO is growing, especially in recent years and its indications remain relatively limited. Finally, the correction angles and gaps obtained in the planning phase have not been tested during surgery, but this was beyond the scope of this study.

## Conclusions

The newly proposed planning method for DLOs, called NM-JL, based on simple measurement tools, showed excellent intra- and inter-rater reliability results and was comparable to the virtual segmentation software method. Obtaining the correction angles and gaps the procedure allows to predict the post-operative JLO. As a result, it is quick, highly adaptable, and reproducible on any DICOM viewer, saving time and costs of dedicated planning software.

### Supplementary Information

Below is the link to the electronic supplementary material.

Supplementary file1 (DOCX 20 KB)

Supplementary file2 (DOCX 25 KB)

## Data Availability

The dataset analysed in this study is available from the corresponding author on reasonable request.

## Abbreviations

HTO: High tibial osteotomy
DFO: Distal femoral osteotomy
JLO: Joint line obliquity
DLO: Double-level osteotomy
AP: Anteroposterior
NM-JL: New Mikulicz joint line
LDFA: Distal lateral femoral angle
MPTA: Proximal medial tibial angle
HKA: Hip–knee–ankle angle
JLCA: Joint line convergence angle
cm: Centimetres
m: Meters
DICOM: Digital imaging and communication in medicine
mm: Millimetres
VSS: Virtual segmentation software
ICC: Intraclass correlation coefficient
CI: Confidence interval
r: Correlation coefficient
LoA: Limits of agreement
SD: Standard deviation
OASIS: Computer-assisted biomechanical analysis software
M-JL: Mikulicz joint line angle
BMI: Body mass index
CORA: Center of rotation of angulation
