# Supplementary material for: A novel geometrical planning method to restore knee joint obliquity in double-level osteotomies
Source: Arch Orthop Trauma Surg. 2023 Jul 28;143(11):6685–93. doi: 10.1007/s00402-023-04997-6 (PMC10541832; doi:10.1007/s00402-023-04997-6)
Supplement: Supplementary file 1 — Supplementary file1 (DOCX 20 KB) [file 402_2023_4997_MOESM1_ESM.docx]

**Additional File 1:** The intraclass correlation coefficient (ICC), two-way random model, with absolute and agreement: 2.1) New Mikulicz Joint Line (NMJL) method; 2.2) Virtual Segmentation Software (VSS) method.

| 2.1) NMJL method (Intra-rater variability and Inter-rater variability) | | | | | | | | |
| --- | --- | --- | --- | --- | --- | --- | --- | --- |
|  |  |  |  |  |  |  |  |  |
|  | **Intra-rater variability** | **ICC** | **CI** |  |  | **Inter-rater variability** | **ICC** | **CI** |
| **Rater A** | A vs B | 0.9902 | 0.9770 to 0.9958 |  | **Rater A  vs Rater B** | A1 vs A | 0.9966 | 0.9920 to 0.9985 |
|  | E vs F | 09975 | 0.9940 to 0.9989 |  |  | B2 vs B | 0.9872 | 0.9665 to 0.9948 |
|  | I vs L | 0.978 | 0.9488 to 0.9906 |  |  | E5 vs E | 0.998 | 0.9954 to 0.9992 |
|  | O vs P | 0.9991 | 0.9978 to 0.9996 |  |  | F6 vs F | 0.9975 | 0.9942 to 0.9989 |
| **Rater B** | A1 vs B2 | 0.9864 | 0.9683 to 0.9942 |  |  | I9 vs I | 0.9838 | 0.9621 to 0.9931 |
|  | E5 vs F6 | 0.998 | 0.9947 to 0.9992 |  |  | L10 vs L | 0.9831 | 0.9604 to 0.9928 |
|  | I9 vs L10 | 0.9777 | 0.9481 to 0.9905 |  |  | O13 vs O | 0.9971 | 0.9933 to 0.9988 |
|  | O13 vs P14 | 0.9978 | 0.9937 to 0.9991 |  |  | P14 vs P | 0.9982 | 0.9958 to 0.9992 |
|  |  |  |  |  |  |  |  |  |
| 2.2) VSS method (Intra-rater variability and Inter-rater variability) | | | | | | | | |
|  | | | | | | | | |
|  | **Intra-rater variability** | **ICC** | **CI** |  |  | **Inter-rater variability** | **ICC** | **CI** |
| **Rater A** | C vs D | 0.9993 | 0.9982 to 0.9997 |  | **Rater A  vs Rater B** | C vs C3 | 0.9995 | 0.9988 to 0.9998 |
|  | G vs H | 0.9968 | 0.9925 to 0.9986 |  |  | D vs D4 | 0.9975 | 0.9941 to 0.9989 |
|  | M vs N | 0.9952 | 0.9888 to 0.9980 |  |  | G vs G7 | 0.9985 | 0.9965 to 0.9994 |
|  | Q vs R | 0.9975 | 0.9943 to 0.9990 |  |  | H vs H8 | 0.9983 | 0.9947 to 0.9993 |
| **Rater B** | C3 vs D4 | 0.9967 | 0.9922 to 0.9986 |  |  | M vs M11 | 0.9973 | 0.9937 to 0.9988 |
|  | G7 vs H8 | 0.9988 | 0.9970 to 0.9995 |  |  | N vs N12 | 0.9943 | 0.9867 to 0.9976 |
|  | M11 vs N12 | 0.9967 | 0.9923 to 0.9986 |  |  | Q vs Q15 | 0.9988 | 0.9971 to 0.9995 |
|  | Q15 vs R16 | 0.9975 | 0.9916 to 0.9991 |  |  | R vs R16 | 0.9984 | 0.9962 to 0.9993 |

ICC: Intraclass-correlation-coefficient; CI: confidence interval; New Mikulicz joint line (NMJL) method; Virtual segmentation sotware (VSS) method; A: Rater A, Femoral correction angle, NMJL method at time zero; B: Rater A, Femoral correction angle, NMJL method after 30 days; C: Rater A, Femoral correction angle, VSS method after 60 days; D: Rater A, Femoral correction angle, VSS method after 90 days; E: Rater A, Millimetre closure gap at femoral level, NMJL method at time zero; F: Rater A, Millimetre closure gap at femoral level, NMJL method after 30 days; G: Rater A, Millimetre closure gap at femoral level, VSS method after 60 days; H: Rater A, Millimetre closure gap at femoral level, VSS method after 90 days; I: Rater A, Tibial correction angle, NMJL method at time zero; L: Rater A, Tibial correction angle, NMJL method after 30 days; M: Rater A, Tibial correction angle, VSS method after 60 days; N: Rate A, Tibial correction angle, VSS method after 90 days; O: Rater A, Millimetre open gap at tibial level, NMJL method at time zero; P: Rater A, Millimetre open gap at tibial level, NMJL method after 30 days; Q: Rater A, Millimetre open gap at tibial level, VSS method after 60 days; R: Rater A, Millimetre open gap at tibial level, VSS method after 90 days; A1: Rater B, Femoral correction angle, NMJL method; B2: Rater B, Femoral correction angle, NMJL method after 30 days; C3: Rater B, Femoral correction angle, VSS method after 60 days; D4: Rater B, Femoral correction angle, VSS method after 90 days; E5: Rater B, Millimetre closure gap at femoral level, NMJL method at time zero; F6: Rater B, Millimetre closure gap at femoral level, NMJL method after 30 days; G7: Rater B, Millimetre closure gap at femoral level, VSS method after 60 days; H8: Rater B, Millimetre closure gap at femoral level, VSS method after 90 days; I9: Rater B, Tibial correction angle, NMJL method at time zero; L10: Rater B, Tibial correction angle, NMJL method after 30 days; M11: Rater B, Tibial correction angle, VSS method after 60 days; N12: Rater B, Tibial correction angle, VSS method after 90 days; O13: Rater B, Millimetre open gap at tibial level, NMJL method at time zero; P14: Rater B, Millimetre open gap at tibial level, NMJL method after 30 days; Q15: Rater B, Millimetre open gap at tibial level, VSS method after 60 days; R16: Rater B, Millimetre open gap at tibial level, VSS method after 90 days.
