# Supplementary material for: A novel geometrical planning method to restore knee joint obliquity in double-level osteotomies
Source: Arch Orthop Trauma Surg. 2023 Jul 28;143(11):6685–93. doi: 10.1007/s00402-023-04997-6 (PMC10541832; doi:10.1007/s00402-023-04997-6)
Supplement: Supplementary file 2 — Supplementary file2 (DOCX 25 KB) [file 402_2023_4997_MOESM2_ESM.docx]

**Additional File 2.** Planning method comparison. New Mikulicz joint line (NMJL) method versus Virtual segmentation software (VSS) method: A) Pearson analysis; B) Bland-Altman analysis.

| 3.1) Pearson analysis | | | | | | | | | | |
| --- | --- | --- | --- | --- | --- | --- | --- | --- | --- | --- |
|  |  |  |  |  |  |  |  |  |  |  |
|  | **Method  comparison** | **r** | **P** | **CI** |  |  | **Method  comparison** | **r** | **P** | **CI** |
| **Rater A** | A vs C | 0.9896 | <0.0001 | 0.9751 to 0.9956 |  | **Rater A** | B vs D | 0.9872 | <0.0001 | 0.9695 to 0.9947 |
|  | E vs G | 0.9943 | <0.0001 | 0.9856 to 0.9975 |  |  | F vs H | 0.9944 | <0.0001 | 0.9865 to 0.9976 |
|  | I vs M | 0.9912 | <0.0001 | 0.9784 to 0.9962 |  |  | L vs N | 0.9872 | <0.0001 | 0.9695 to 0.9947 |
|  | O vs Q | 0.9924 | <0.0001 | 0.9819 to 0.9968 |  |  | P vs R | 0.9966 | <0.0001 | 0.9918 to 0.9986 |
| **Rater B** | A1 vs C3 | 0.9964 | <0.0001 | 0.9913 to 0.9985 |  | **Rater B** | B2 vs D4 | 0.9869 | <0.0001 | 0.9688 to 0.9945 |
|  | E5 vs G7 | 0.9987 | <0.0001 | 0.9969 to 0.9995 |  |  | F6 vs H8 | 0.9973 | <0.0001 | 0.9934 to 0.9989 |
|  | I9 vs M11 | 0.9906 | <0.0001 | 0.9775 to 0.9961 |  |  | L10 vs N12 | 0.9869 | <0.0001 | 0.9688 to 0.9945 |
|  | O13 vs Q15 | 0.9983 | <0.0001 | 0.9958 to 0.9993 |  |  | P14 vs R16 | 0.9971 | <0.0001 | 0.9932 to 0.9988 |
|  |  |  |  |  |  |  |  |  |  |  |
| 3.2) Bland-Altman analysis | | | | | | | | | | |
|  |  |  |  |  |  |  |  |  |  |  |
|  | **Method  comparison** | **P** | **CI upper limit** | **CI lower limit** |  |  | **Method  comparison** | **P** | **CI upper limit** | **CI lower limit** |
| **Rater A** | A vs C | 0.0004 | 0.08852 to 0.4839 | 0.9448 to -0,5494 |  | **Rater A** | B vs D | 0.0001 | 0.0650 to 0.5228 | -1.1315 to -0.6737 |
|  | E vs G | 0.0419 | 0.1907 to 0.5672 | -0.7933 to -0.4168 |  |  | F vs H | 0.0265 | 0.1691 to 0.5242 | -0.7590 to -0.4038 |
|  | I vs M | 0.0001 | -0.7420 to -0.4420 | 0.0420 to 0.3420 |  |  | L vs N | 0.0078 | 0.1323 to 0.4635 | -0.7330 to -0.4019 |
|  | O vs Q | 0.0208 | -0.8113 to -0.4344 | 0.1736 to 0.5504 |  |  | P vs R | 0.0384 | 0.1280 to 0.3836 | -0.5402 to -0.2845 |
| **Rater B** | A1 vs C3 | <0.0001 | -0.01515 to 0.2086 | -0.5999 to -0.3762 |  | **Rater B** | B2 vs D4 | 0.0339 | 0.2247 to 0.6810 | -0.9680 to -0.5116 |
|  | E5 vs G7 | 0.0127 | 0.07422 to 0.2471 | -0.3775 to -0.2047 |  |  | F6 vs H8 | 0.0159 | 0.1115 to 0.3630 | -0.5456 to -0.2941 |
|  | I9 vs M11 | 0.0001 | 0.03306 to 0.3328 | -0.7502 to -0.4505 |  |  | L10 vs N12 | 0.0122 | 0.1516 to 0.5066 | -0.7761 to -0.4211 |
|  | O13 vs Q15 | 0.0272 | 0.08839 to 0.2734 | -0.3952 to -0.2101 |  |  | P14 vs R16 | 0.0207 | 0.1040 to 0.3299 | -0.4865 to -0.2605 |

P: p.value; r: Pearson correlation coefficient; CI: confidence interval; New Mikulicz joint line (NMJL) method; Virtual segmentation sotware (VSS) method; A: Rater A, Femoral correction angle, NMJL method at time zero; B: Rater A, Femoral correction angle, NMJL method after 30 days; C: Rater A, Femoral correction angle, VSS method after 60 days; D: Rater A, Femoral correction angle, VSS method after 90 days; E: Rater A, Millimetre closure gap at femoral level, NMJL method at time zero; F: Rater A, Millimetre closure gap at femoral level, NMJL method after 30 days; G: Rater A, Millimetre closure gap at femoral level, VSS method after 60 days; H: Rater A, Millimetre closure gap at femoral level, VSS method after 90 days; I: Rater A, Tibial correction angle, NMJL method at time zero; L: Rater A, Tibial correction angle, NMJL method after 30 days; M: Rater A, Tibial correction angle, VSS method after 60 days; N: Rate A, Tibial correction angle, VSS method after 90 days; O: Rater A, Millimetre open gap at tibial level, NMJL method at time zero; P: Rater A, Millimetre open gap at tibial level, NMJL method after 30 days; Q: Rater A, Millimetre open gap at tibial level, VSS method after 60 days; R: Rater A, Millimetre open gap at tibial level, VSS method after 90 days; A1: Rater B, Femoral correction angle, NMJL method at time zero; B2: Rater B, Femoral correction angle, NMJL method after 30 days; C3: Rater B, Femoral correction angle, VSS method after 60 days; D4: Rater B, Femoral correction angle, VSS method after 90 days; E5: Rater B, Millimetre closure gap at femoral level, NMJL method at time zero; F6: Rater B, Millimetre closure gap at femoral level, NMJL method after 30 days; G7: Rater B, Millimetre closure gap at femoral level, VSS method after 60 days; H8: Rater B, Millimetre closure gap at femoral level, VSS method after 90 days; I9: Rater B, Tibial correction angle, NMJL method at time zero; L10: Rater B, Tibial correction angle, NMJL method after 30 days; M11: Rater B, Tibial correction angle, VSS method after 60 days; N12: Rater B, Tibial correction angle, VSS method after 90 days; O13: Rater B, Millimetre open gap at tibial level, NMJL method at time zero; P14: Rater B, Millimetre open gap at tibial level, NMJL method after 30 days; Q15: Rater B, Millimetre open gap at tibial level, VSS method after 60 days; R16: Rater B, Millimetre open gap at tibial level, VSS method after 90 days.
